# Sleep Bruxism and Sleep Structure in Comorbid Insomnia and Obstructive Sleep Apnea (COMISA) Syndrome: A Polysomnographic Study

**DOI:** 10.3390/jcm13113154

**Published:** 2024-05-28

**Authors:** Bartlomiej Blaszczyk, Miguel Meira e Cruz, Marta Waliszewska-Prosol, Mieszko Wieckiewicz, Dorian Nowacki, Justyna Kanclerska, Gabriella Lachowicz, Anna Wojakowska, Monika Michalek-Zrabkowska, Jakub Przegralek, Joanna Smardz, Katarzyna Antosz, Grzegorz Mazur, Helena Martynowicz

**Affiliations:** 1 Student Research Club No K133, Faculty of Medicine, Wroclaw Medical University, 213 Borowska St., 50-556 Wroclaw, Poland; 2 Sleep Unit, Centro Cardiovascular da Universidade de Lisboa, Department of Cardiology, Lisbon School of Medicine, 1649-028 Lisbon, Portugal; 3 Department of Neurology, Wroclaw Medical University, 213 Borowska St., 50-556 Wroclaw, Poland; 4 Department of Experimental Dentistry, Wroclaw Medical University, 26 Krakowska St., 50-425 Wroclaw, Poland; 5 Department of Human Nutrition, Wroclaw University of Environmental and Life Sciences, 37 Chelmonskiego St., 51-630 Wroclaw, Poland; 6 Department and Clinic of Internal Medicine, Occupational Diseases, Hypertension and Clinical Oncology, Wroclaw Medical University, 213 Borowska St., 50-556 Wroclaw, Poland

**Keywords:** COMISA, OSA, PSG, bradycardia, SB, sleep quality

## Abstract

**Introduction:** Comorbid insomnia and obstructive sleep apnea (COMISA) is not a well-identified sleep disorder, despite having a significant impact on health. This study investigates the relationship between sleep bruxism (SB) and sleep architecture in patients with COMISA, obstructive sleep apnea (OSA), and in those without any sleep disorders. **Methods**: 119 patients were included in the study and divided into three groups: OSA, COMISA, and a control group. Polysomnographic (PSG) examination provided parameters related to sleep architecture, OSA, and characteristics of SB. **Results**: The bruxism episode index (BEI) and other SB parameters were not found to be statistically different between the three groups (*p* > 0.05). There was no statistical difference in measured sleep architecture between the COMISA and OSA groups (*p* > 0.05). In comparison to the control group, participants in the COMISA group were found to have an increased apnea–hypopnea index (AHI), oxygen desaturation index (ODI), respiratory disturbance index (RDI), all arousals (AA), and respiratory arousals (RA) (*p* < 0.05). Among COMISA patients, AA and RA were shown to have a positive linear correlation with the number of bradycardia events per hour (r = 0.49, r = 0.48, *p* < 0.05). **Conclusions**: SB does not occur in patients with COMISA more frequently than in patients with OSA or those without any sleep disorders. PSG parameters are not specific for COMISA; therefore, in order to differentiate this disorder from OSA alone, a comprehensive patient assessment has to be performed.

## 1. Introduction

The term “COMISA”, which refers to concurrent insomnia and obstructive sleep apnea (OSA), is a relatively new term, having first been mentioned in the literature only in 2017 [1]. It is worth noting that the first study describing the co-existence of insomnia and OSA had been published almost 45 years earlier, in 1973 [2]. Unfortunately, despite the high prevalence of OSA coexisting with insomnia, there have been very few studies over the years regarding this subject [3]. OSA is a sleep disorder characterized by a repetitive collapse of the upper airway, leading to partial cessation or a complete block of airflow during sleep [4]. This leads to arterial oxygen desaturation and changes in intrathoracic pressure, ultimately generating nocturnal arousals; OSA affects about 9% to 38% of the population [5,6]. Clinical symptoms include snoring, fatigue, excessive daytime sleepiness, and a reduced quality of life [7,8]. Insomnia is the second most commonly occurring sleep disorder worldwide and consists of problems in initiating or maintaining sleep, despite having optimal conditions and opportunities for sleep [9]. Thirty percent of the population will experience symptoms of insomnia at least once in their lifetime. Waking up too early and experiencing poor quality of sleep are symptoms that are also indicative of insomnia [10].

After researchers’ attention had been drawn to the occurrence of OSA with insomnia and after further research had been conducted, a number of discoveries were published. First of all, the prevalence of COMISA continues to be high, varying from 29.2% to 38%, and up to as many as 50% of participants in certain studies [11,12]. Patients have reported more intense symptoms (such as increased daytime sleepiness or daytime impairments, for instance, fatigue, memory difficulties, and decreased mood) and an overall reduced quality of life and sleep compared to patients suffering from either insomnia or OSA alone [13,14]. Males have been found to be more commonly affected by the decreased quality of life and fatigue than females. Furthermore, these characteristics have been found to occur more frequently in posttraumatic stress disorder (PTSD) patients, military personnel, or war veterans [15,16,17]. Additionally, COMISA has been found to significantly increase the risk of cardiovascular disease (CVD), hypertension, and mortality. It has also been associated with depression, stress, anxiety, or diabetes type 2 more so than OSA or insomnia alone [18,19,20,21].

However, in the literature, the relationship between COMISA and sleep bruxism (SB) has yet to be explored. SB is a condition characterized by rhythmic (phasic) or nonrhythmic (tonic) masticatory muscle activity during sleep that ultimately results in the grinding or clenching of teeth and/or bracing or thrusting of the mandible [22]. Bruxism has been estimated to affect around 13% of the population; however, it is not considered to be a movement or sleep disorder in otherwise healthy individuals [23]. SB has been found to occur more frequently in patients with particular sleep disorders, such as OSA or restless leg syndrome (RLS) [24,25]. Additionally, several studies have shown a positive connection between OSA and SB [26,27]; however, OSA has not always been linked with SB [28]. There exists a theoretical “temporal” mechanism against OSA in concomitant SB and OSA where, during bruxism episodes, mandible protrusion maintains the patency of the upper airway tract [29,30]. COMISA syndrome consists of OSA and insomnia alone but it has not been estimated as to whether SB could have any connection in COMISA using a gold standard method to diagnose sleep disorders such as polysomnography with video-recording [31].

Therefore, to examine the relationship between SB and COMISA syndrome using valid methods, the objective of our study was to investigate the potential characteristics of SB in patients with COMISA syndrome vs. an OSA group vs. patients without any sleep disorders. Additionally, the full sleep architecture from the PSG examination was compared between these groups.

## 2. Materials and Methods

**Figure 1:**
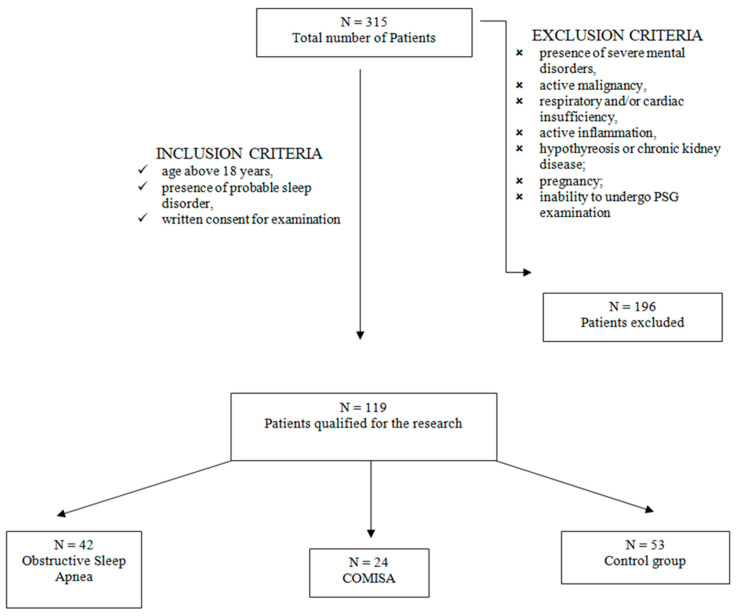
Flowchart for accepting patients into the study.

Qualified researchers from the Sleep Laboratory of the Department and Clinic of Internal Medicine, Occupational Diseases, Hypertension and Clinical Oncology at Wroclaw Medical University, Poland, recruited 119 patients for the study. Among these patients, 73 were male and 60 female. The patients were divided into three groups: an obstructive sleep apnea group consisting of 42 patients, a COMISA group including 24 patients, and a control group (patients with no sleep disorders) with 53 subjects. (Figure 1).

This study was in accordance with the Declaration of Helsinki. Before participating in the examination, all patients had signed an informed written consent form. The study obtained approval from the Ethical Committee at Wroclaw Medical University (no. KB-523/2021). The research is registered at clinicaltrials.gov, and the examination reference number is NCT04937036.

### 2.1. Study Participants

In this present study, patients were admitted to the Sleep Laboratory at the Department and Clinic of Internal Medicine, Occupational Diseases, Hypertension, and Clinical Oncology at Wroclaw Medical University in Poland for video PSG examination. Patients that were included in the study were 18 years of age or older, had a probable sleep disorder, and had signed an informed consent form for the examination.

Among the exclusion criteria was the presence of severe mental illness, active malignant process, cardiac and/or respiratory disease, and active inflammation, as well as other severe metabolic diseases. Also, pregnant women were excluded from the study as were those unable to undergo polysomnographic examination. Qualified medical doctors divided the participants into the above-mentioned study groups on the basis of detailed criteria. The presence of insomnia in COMISA was diagnosed according to the fifth edition criteria of the Diagnostic and Statistical Manual of Mental Disorders (DSM-5) and the International Classification of Sleep Disorders (3rd edition, ICSD-3). The criteria specify that there is the presence of a nocturnal sleep disturbance (criterion A) and a daytime-related impairment (criterion B). Furthermore, there is an occurrence of the sleep disorder for at least three nights a week for a minimum three-month period of time, thus enabling a diagnosis of a clinically relevant disorder [32].

Regarding the treatment of insomnia, there is a fast-screening tool, the Insomnia Severity Index (ISI), which is used by researchers as an outcome measure. The ISI contains seven factors that assess the difficulties of sleep maintenance (both nocturnal and early morning awakenings) and sleep onset. These include satisfaction with the current sleep pattern, interference with daily functioning, noticeable impairment (by a second or third party) that can be attributed to the sleep problem, and the stage of distress or any concern that can be caused by the sleep problem. Each factor is scored on a scale from 0 to 4 with the total maximum score being 28 points. A higher score can indicate more severe insomnia [33]. Categories of insomnia are defined based on ISI scores as follows: no insomnia (0–7) and mild to severe insomnia (8–28) [34].

### 2.2. Polysomnographic Examination

Polysomnograms were performed in accordance with the standard sleep scoring criteria of the American Academy of Sleep Medicine (AASM, 2013) using Nox-A1 (Nox Medical, Reykjavík, Iceland). We recorded the bioelectrical activity of the brain with the use of electroencephalography (EEG), whereby shifts changes in the EEG to a higher frequency (frequencies > 16 Hz) lasting ≥3 s occurring after ≥10 s of stable sleep were defined as cortical arousals [35]. Electro-oculography (EOG) was used to record eye movement. In addition, the airflow was recorded with a nasal pressure sensor; inductive plethysmography was used for chest and abdominal movements and an electromyogram showed the muscular tension from the tibial electrodes. Moreover, the body position of the patient was recorded and lateral masseter electromyography (EMC) was also performed.

After examination, a qualified physician (author HM) from the Sleep Laboratory, Wroclaw Medical University, Poland, manually analyzed the collected data on the Noxturnal system (version 2.6). Scoring of the sleep disorders was performed in accordance with the AASM guidelines. Participants assigned to the OSA group were also selected according to AASM guidelines.

According to the International Classification of Sleep Disorders (3rd edition, ICSD-3), the definition of OSA is having a PSG-determined obstructive respiratory disturbance Index (RDI) ≥ five events/h that is associated with the typical symptoms of OSA. There can be a presence of daytime sleepiness, fatigue, insomnia, ineffective sleep, awakenings with a choking sensation, witnessed apneas, or loud snoring. When the obstructive RDI score is higher than 15 events/h, the OSA is recognized even with the absence of symptoms [36]. In addition to this, rhythmic masseter muscle activity (RMMA) was assessed according to the recorded audio sounds with the electromyography (EMG) of the bilateral masseters and chin region using surface electrodes. After inspection of the skin and making sure that it is in a dry and clean location that has no wounds or abrasions, the skin was cleaned with water and abrasive skin-prepping gel. If the skin was very oily, wipes with alcohol were used. The electrodes were applied to the skin using paste ensuring biocompatibility and electrical contact. When diagnosing the SB, an amplitude at least double that of the background EMG activity was required. Pauses between the EMG bursts that indicate the same SB episode should not be longer than three seconds. Additionally, at least two audible tooth-grinding episodes that coexisted with the EMG bursts were required to establish the bruxism diagnosis. In accordance with the ASM guidelines, bruxism episodes were classified into phasic, tonic, and mixed forms. The bruxism episode index (BEI) was obtained based on the number of bruxism events per hour of sleep. The intensity of the sleep bruxism was divided into insignificant (BEI < 2), mild or moderate (BEI = 2–4), or severe (BEI > 4).

### 2.3. Statistical Analysis of the Results

The statistical analysis was performed with Statistica 13.3 (Statsoft, Cracow, Poland). The obtained data are presented as means and standard deviations. To determine any correlations, the Spearman rank test was used. Variance analysis (ANOVA) followed by a post hoc test was used for all parametric data and Kruskal–Wallis ANOVA for the remaining data, i.e., non-parametric data. A *p* value of <0.05 was considered statistically significant. The differences between all groups, i.e., COMISA vs. OSA, COMISA vs. control, and OSA vs. Control, were analyzed in the study but, according to the study objectives, only results from comparisons between COMISA and OSA and the control group are shown.

## 3. Results

A total of 119 patients were enrolled in the study, 59 males and 60 females, with an average age of 47.0 ± 6.8 years. The OSA group comprised 42 patients, with a mean age of 52.8 ± 14.4 years. Twenty-four participants were included in the COMISA group, with an average age of 59.8 ± 13.8 years. Finally, 53 patients made up the control group, with a mean age of 36.0 ± 12.9 years. BMI was found to be highest in the COMISA group (31.7 ± 5.0 kg/m^2^) and lowest in the control group (23.9 ± 4.1 kg/m^2^). OSA patients had an average BMI of 29.4 ± 4.1 kg/m^2^. The fact that participants in the COMISA group had a higher BMI than patients in the control group was found to be statistically significant (*p* < 0.05). These data are presented in Table 1.

In terms of sleep architecture, the COMISA group did not differ significantly from the OSA group (*p* > 0.05). However, in comparison to participants in the control group, patients with COMISA were found to have higher respiratory parameters such as AHI, ODI, RDI, apneas, hypopneas per hour, all arousals (AA), and respiratory arousals (RA) with snoring. This was found to be statistically significant (*p* < 0.05). PSG parameters that were assessed in the examined groups are presented in Table 2.

A Bruxism episode index (BEI) > 2 was observed in 80 out of the 119 patients (67%), of which 36 were male (61%) and 41 were female (72%) participants. In total, 27 OSA patients (64%), 13 COMISA (54%), and 40 control patients (71%) received an SB diagnosis based on the aforementioned BEI value. BEI and other SB parameters, however, were not found to be statistically different between COMISA and the comparator groups (*p* > 0.05). The results are presented in Table 3.

## 4. Discussion

The main result of this study is that SB does not occur more frequently in patients with COMISA. There is only one existing study that reports the lack of an association between SB and COMISA in comparison to OSA alone [37]. The results of that study are consistent with our observations. However, in those studies, SB diagnosis was based on self-reported symptoms of bruxism and did not include a control group that would allow for a comparison of results [37]. Due to the limited number of publications addressing the relationship between bruxism and COMISA, it is challenging to discuss our results in relation to those mentioned in other studies. There is a need to conduct further research on this topic. Comparing our observations to individual components of COMISA, such as OSA, it appears that patients with OSA often have fewer SB episodes [28,38,39]. This relationship appears to be so significant that some authors have even referred to SB as a “protective factor” against the development of OSA [32]. Articles found in our analysis of the existing literature have not shown a positive correlation between AHI and BEI in OSA [40,41,42]. Our COMISA group with an increased AHI value also lacks a correlation between respiratory disturbances and BEI, which could indicate that COMISA may be a protective factor against the development of SB. However, we did not attempt a categorization of COMISA according to OSA severity; therefore, further studies are required to explain the relationship between the AHI value and SB.

Previous studies examining patients with COMISA have presented inconclusive results on sleep structure. In our article, there were no differences in sleep structure between COMISA and OSA patients. However, we did observe that, in comparison to the control group, respiratory PSG parameters characteristic for OSA (such as AHI, ODI, RDI, respiratory issues, or apnea arousals) were significantly higher than in the COMISA group. The opposite respiratory results were obtained in a study conducted by Mysliwiec et al. [43], whose OSA patients had the highest levels for the above breathing parameters, followed by the COMISA participants. In other studies, only AHI differed between groups and, once again, this was higher in OSA than the COMISA groups [16]. Wulterkens et al. [44] defined the difference in COMISA, OSA, and insomnia with additional “insomnia” features, such as increased WASO or Sleep Onset Latency without changes in respiratory parameters [45]. Some articles have also noticed changes in both insomnia and respiratory parameters [43]. We only observed significant differences in respiratory parameters when comparing the COMISA and OSA patients to our control group. Unfortunately, the previously mentioned studies did not contain control groups that would allow for a direct comparison of our results. Our analysis of the existing literature found that PSG variables such as total sleep time (TST) and sleep efficiency were found to be different in certain groups of patients [16,45,46]. On the other hand, we did not observe these correlations in our study because these differences found in other research may influence group profiles such that they consist of only insomnia patients who were not present in our study. We would like to emphasize that some of the cited studies were conducted among soldiers and PTSD patients, as opposed to our study, which did not include patients with these characteristics. These features could influence the final results. It is also worth mentioning that concomitant SB could change sleep architecture and may play a role in changing particular parameters [47].

The presented study has a few limitations. First, the COMISA group consisted of only 24 patients; therefore, future studies should include a larger group of participants to avoid the potential risk of bias. Our study also did not include a group of patients suffering from insomnia alone, thus restricting our ability to provide a comparison between COMISA and insomnia patients. For these reasons, our results may be different from studies related to this topic. It is also worth mentioning that the PSG examination was conducted without an adaptive night in the hospital; therefore, the obtained PSG results may contain a risk of bias.

## 5. Conclusions

SB does not occur more frequently in COMISA than in OSA or the control group. COMISA did not show specific deviations in PSG parameters in comparison to OSA subjects; however, it was found to cause serious respiratory disturbances when comparing the results to the control group. To distinguish COMISA from OSA, a comprehensive patient assessment needs to be conducted, as PSG alone does not allow for the diagnosis of COMISA.

## Figures and Tables

**Table 1:** Main characteristics of the included patients.

	COMISA (*n* = 24)	OSA (*n* = 42)	*p* Value	Control (*n* = 53)	*p* Value
Age	59.8 ± 13.8	52.8 ± 14.4	*p* > 0.05	36.0 ± 13.00	*p* < 0.05
BMI	31.8 ± 5.0	29.4 ± 4.1	*p* > 0.05	23.9 ± 4.0	*p* < 0.05
Number (%) of patients of particular group with Bruxism Episodes/h ≥ 2	13 (54%)	27 (64%)	-----	40 (75%)	------

**Table 2:** PSG parameters among COMISA, OSA, and control group. The *p* value was presented as a comparison between patients diagnosed with COMISA and other groups.

PSG PARAMETERS	COMISA (*n* = 24)		OSA (*n* = 42)		*p* Value	Control (*n* = 53)		*p* Value
	Average	SD	Average	SD		Average	SD	
Sleep Efficiency [%]	79.0	16.6	80.6	12.4	*p* > 0.05	83.3	15.7	*p* > 0.05
N1% of TST	8.5	7.7	7.0	5.6	*p* > 0.05	4.6	2.6	*p* > 0.05
N2% of TST	46.3	11.4	45.7	11.6	*p* > 0.05	52.6	26.2	*p* > 0.05
N3% of TST	25.2	9.2	25.3	9.2	*p* > 0.05	29.0	37.5	*p* > 0.05
REM% of TST	20.1	9.9	21.9	8.2	*p* > 0.05	24.5	11.0	*p* > 0.05
Sleep Latency [m]	24.0	38.1	16.9	14.0	*p* > 0.05	14.8	10.9	*p* > 0.05
REM Latency [m]	104.01	83.1	91.2	77.1	*p* > 0.05	108.4	82.0	*p* > 0.05
Wake After Sleep Onset [m]	81.9	80.6	72.3	56.2	*p* > 0.05	52.6	58.0	*p* < 0.05
AHI/h	43.2	30.1	23.7	15.2	*p* > 0.05	2.0	1.3	*p* < 0.05
ODI/h	39.6	28.0	21.7	14.4	*p* > 0.05	2.3	1.6	*p* < 0.05
Snore%	33.4	20.6	29.8	20.2	*p* > 0.05	6.3	12.4	*p* < 0.05
RDI/h	43.5	29.9	24.2	15.3	*p* > 0.05	2.3	1.5	*p* < 0.05
Apneas/h	25.4	24.4	11.3	12.4	*p* > 0.05	0.8	0.7	*p* < 0.05
Hypopneas/h	17.8	14.5	12.4	6.7	*p* > 0.05	1.2	1.1	*p* < 0.05
AA/h	10.6	10.4	7.6	5.8	*p* > 0.05	4.7	2.8	*p* < 0.05
RA/h	7.8	10.5	3.8	5.5	*p* > 0.05	0.2	0.4	*p* < 0.05
Av SpO_2_%	91.3	2.0	92.4	1.8	*p* > 0.05	94.8	1.4	*p* > 0.05
Min SpO_2_	74.4	10.3	80.9	6.4	*p* > 0.05	87.9	5.2	*p* < 0.05
SpO_2_ Duration < 90%	21.2	19.0	12.0	17.9	*p* > 0.05	1.8	4.9	*p* < 0.05
PLMS/h	8.5	15.3	11.5	26.2	*p* < 0.05	5.7	8.9	*p* < 0.05
Bradycardia/h	0.1	0.2	0.9	2.6	*p* > 0.05	0.3	1.3	*p* > 0.05
Tachycardia/h	1.7	6.1	0.2	0.4	*p* > 0.05	0.2	0.7	*p* > 0.05

Abbreviations: PSG—polysomnography; COMISA—co-occurring insomnia and obstructive sleep apnea; OSA—obstructive sleep apnea; TST—total sleep time; N1—non-rapid eye movement sleep stage 1; N2—non-rapid eye movement sleep stage 2; N3—non-rapid eye movement sleep stage 3; REM—rapid eye movement; AHI/h—apnea–hypopnea index per hour; ODI/h—oxygen desaturation index per hour; RDI/h—respiratory disturbance index per hour; AA/h—all arousals; RA/h—respiratory arousals; SpO_2_—oxygen saturation; Av—average; Min—minimal; PLMS—periodic limb movement syndrome index.

**Table 3:** Bruxism parameters measured during polysomnography studies in patients. *p* value was calculated as a comparison between COMISA patients and other individual groups.

PSG PARAMETRS	COMISA (*n* = 24)		OSA (*n* = 42)		*p* Value	Control (*n* = 53)		*p* Value
	Average	SD	Average	SD		Average	SD	
Bruxism Episodes Index (BEI)	4.8	3.8	3.4	2.4	*p* > 0.05	4.8	3.1	*p* > 0.05
Phasic Episodes/h	2.0	1.5	1.6	1.6	*p* > 0.05	2.0	2.5	*p* > 0.05
Tonic Episodes/h	2.0	1.9	1.2	0.9	*p* > 0.05	2.0	0.8	*p* > 0.05
Mixed Episodes/h	0.7	0.9	0.5	0.5	*p* > 0.05	0.7	0.6	*p* > 0.05
Bruxism Bursts Index	10.8	9.2	10.1	9.7	*p* > 0.05	10.8	17.5	*p* > 0.05

Abbreviations: PSG—polysomnography; COMISA—co-occurring insomnia and obstructive sleep apnea; OSA—obstructive sleep apnea; BEI—Bruxism Episodes Index.

## Data Availability

Dataset available on request from the authors.
